# Pulmonary Artery Dilatation Due to Pressure or Volume Overload in Congenital Heart Disease

**DOI:** 10.3390/jcm13061567

**Published:** 2024-03-09

**Authors:** Monika Kaldararova, Katarina Bobocka, Andrea Kantorova, Erika Drangova, Jana Polakova Mistinova, Filip Klauco, Tereza Hlavata, Adriana Reptova, Tatiana Valkovicova, Iveta Simkova

**Affiliations:** 1 Children’s Cardiac Center, National Institute of Cardiovascular Diseases, Pod Krasnou Horkou 1, 833 48 Bratislava, Slovakia; 2 Department of Adult Congenital Heart Diseases, National Institute of Cardiovascular Diseases and Slovak Medical University, Pod Krasnou Horkou 1, 833 48 Bratislava, Slovakiafilip.klauco@nusch.sk (F.K.); iveta.simkova@nusch.sk (I.S.); 3 Department of Diagnostic and Interventional Radiology, National Institute of Cardiovascular Diseases and Slovak Medical University, Pod Krasnou Horkou 1, 833 48 Bratislava, Slovakia; erika.drangova@nusch.sk (E.D.);

**Keywords:** pulmonary artery diameter, pulmonary artery dilatation, pulmonary artery to ascending aorta ratio, pulmonary arterial hypertension, repaired Tetralogy of Fallot, pulmonary regurgitation

## Abstract

**Background:** Pulmonary artery dilatation is described mostly in association with pulmonary hypertension. **Patients/Methods:** Study analysis: 60 patients with pulmonary arterial hypertension in congenital heart disease (PAH-CHD); 64 with repaired tetralogy of Fallot/pulmonary regurgitation (rTOF/PR); and 80 healthy (NORMAL). Measured were: main pulmonary artery (MPA) diameter and MPA/ascending aorta (Ao asc) ratio, by echocardiography (ECHO) and computer tomography or magnetic resonance imaging (CT/MRI). **Results:** In MPA diameter, significant differences between PAH-CHD, rTOF/PR, and NORMAL were found (median): 37 vs. 27 vs. 21 mm (*p* < 0.0001). In MPA/Ao asc ratio, there was a difference between PAH-CHD and NORMAL (median): 1.3 vs. 0.8 (*p* < 0.0001), but not between rTOF/PR and NORMAL: 0.74 vs. 0.8 (*p* = 0.3). Significant MPA dilatation (>40 mm) was present: in PAH-CHD, 35% (ECHO) and 76.9% (CT/MRI) of patients, while in rTOF/PR, 3.1% (ECHO) and 7.8% (CT/MRI). Severe MPA dilatation (>50 mm) occurred only in PAH-CHD: 16.7% (ECHO) and 31.4% (CT/MRI), while not in rTOF/PR. There was a significant correlation between ECHO and CT/MRI measurements, but ECHO was underestimated in all parameters. **Conclusions:** MPA dilatation due to pressure overload is more frequent and more severe; volume overload also leads to MPA dilatation but is less severe. The MPA/Ao asc ratio is not reliable for MPA dilatation estimation in rTOF/PR.

## 1. Introduction

The morphology and pathophysiology of the pulmonary artery (PA) is an important topic, though it is less frequently analyzed in the literature. Usually, dilatation of the main pulmonary artery (MPA) is considered when the diameter is above 29–30 mm [1].

Pathological factors that are influencing the PA size and can lead to dilatation may be congenital or acquired. From the hemodynamic point of view, the most important pathological factor is the long-term presence of elevated PA pressure, which leads to increased wall shear stress and chronic vascular remodeling [2]. In addition to that, an increased or turbulent PA flow contributes to the vessel dilatation as well. Fibrosis and other interstitial lung processes can also alter the pulmonary vasculature and its compliance, increasing pulmonary resistance. PA atherosclerosis, mural calcifications, genetic or autoimmune disorders, primary vasculopathy, connective tissue disease, or infection can also cause PA wall damage, weakness, or increased fragility and lead to PA dilatation or aneurysm formation [3,4,5].

The most relevant complications that may result from PA dilatation are [6,7,8,9,10,11,12]:

- External compression of the main left coronary artery by the dilated PA;
- PA dissection/rupture (with angina, hemoptysis, tamponade, or hemothorax—according to the site of rupture);
- PA thrombosis (from asymptomatic to severe forms with hemoptysis);
- Airway compression;
- Recurrent laryngeal nerve compression.

These complications and their clinical symptoms may be life-threatening for the patients and may need acute action. On the other hand, these patients are often asymptomatic from PA dilatation for a long time but are at very high risk due to their underlying disease. There are no clear guidelines about the severity of PA dilatation, the cut-off points for any intervention, or the optimal timing for this.

Most of the complications due to PA dilatation are being described in, in case reports, and usually in association with pulmonary hypertension (PH) [9,10,11,12]. It is unclear, though, if only the significantly increased PA pressure is the risk factor or if any of these severe complications can also occur due to other underlying mechanisms of PA dilatation, also in situations with normal PA pressure.

## 2. The Aim of This Study

The aim of our study was to analyze the MPA size and its dilatation in two groups of congenital heart defect patients with different underlying chronic hemodynamic pathologies: 1. PA pressure overload (due to severe PH); and 2. pure PA volume overload (due to “free” pulmonary regurgitation); and compare it with age-matched healthy volunteers.

## 3. Patients and Methods

In this retrospective study, we included adult patients that were regularly followed at our institution between 2008 and 2023:

- Sixty patients with **pulmonary arterial hypertension associated with congenital heart defect (PAH-CHD)** (47 patients with unoperated shunt defects; 10 after surgical or interventional shunt closure, or with small defects; and 3 with congenital portopulmonary PH); all with the long-term (median 15 years) confirmed hemodynamics of severe pulmonary artery pressure overload (with mean pulmonary artery pressure 60 mmHg (median), and pulmonary vascular resistance 9.3 Wood units (median)).
- Sixty-four patients **with a repaired Tetralogy of Fallot with isolated “free” pulmonary regurgitation (rTOF/PR)** had the hemodynamics of pure proximal pulmonary artery volume overload. The median age at complete TOF repair was 5 years, and the median follow-up after repair was 22 years. The PA data analysis was performed at the time period before any reintervention due to pulmonary regurgitation was performed. In this study, patients with Tetralogy of Fallot with absent pulmonary valve syndrome, patients with pulmonary conduit or any other valve implantation during primary complete repair, or patients with any presence of significant (valvar, subvalvar, or supravalvar) pulmonary stenosis or significant pulmonary artery branch deformation during follow-up were not included.
- In this study, 80 age-matched healthy volunteers without any significant cardiac disease, with normal structural and functional findings, without systemic or pulmonary hypertension, without arrhythmia, or any known chronic diseases like diabetes or connective tissue disease **(NORMAL)** were included.

All basic patients’ characteristics are summarized in Table 1, PAH-CHD patients’ characteristics are summarized in Table 2, and rTOF/PR in Table 3.

In all patients analyzed, transthoracic echocardiography (ECHO) was performed with all standard imaging planes [13,14]. Measured were: the main pulmonary artery (MPA) diameter from the parasternal short axis view (measured at midway between the pulmonary artery valve and the PA bifurcation, at end-diastole, using the inner edge to inner edge method); and the ascending aorta (Ao asc) diameter from the parasternal long axis view (measured above the sinotubular junction, at end-diastole, using the leading edge to leading edge method). The MPA/Ao asc ratio was also calculated. All ECHO measurements were obtained during a routine ECHO examination at a regular out-patient visit or retrospectively from the archive database of the Picture Archiving and Communications System (PACS) of our institution. 

Thirty-five patients with PAH-CHD and all 64 patients with rTOF/PR were also evaluated by computer tomography and/or magnetic resonance imaging (CT/MRI). Measured were MPA and Ao asc diameters, both from a standard axial plane, using the inner edge to inner edge measurement method [15,16,17,18]. The MPA/Ao asc ratio was also calculated. All CT/MRI measurements were obtained retrospectively from the PACS archive database of our institution, from examinations that were performed according to the standard protocol and indication criteria during the patient’s follow-up, outside the aim of this study. CT/MRI were not performed on NORMAL subjects. In the same patient, the time span between ECHO and CT/MRI examinations was not more than 2 weeks. 

In all the analyzed parameters, 3 measurements were obtained, and the mean value was used. The obtained data were plotted against patients’ age and body surface area (BSA). 

Performed was the comparison of PAH-CHD and rTOF/PR groups, and in ECHO measurements also the comparison with the NORMAL group. The same parameters were obtained by ECHO and CT/MRI. 

**Figure 1:**
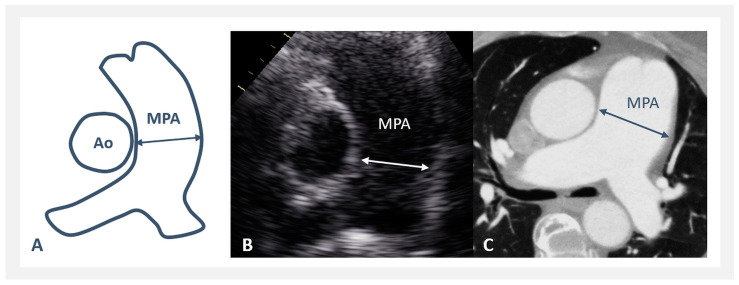
Pulmonary artery measurement: (**A**) scheme; (**B**) by echocardiography; and (**C**) by computer tomography (MPA—main pulmonary artery; Ao—aorta).

Dilated MPA was considered when the measured diameter was >30 mm; significant dilatation was considered MPA diameter >40 mm; and severe dilatation >50 mm, both in ECHO as well as CT/MRI measurements (Figure 1). Pathological MPA/Ao asc ratio was considered >1, both in ECHO and CT/MRI calculations, and severe dilatation with the MPA/Ao asc ratio >1.5.

### Statistical Analysis

The Student’s *t*-test or one-way analysis of variance test, for normally distributed data and the Wilcoxon/Kruskal–Wallis test for non-parametric data. In the case of nominal data, contingency tables were used. These comparisons and the logistic regression analyses were performed using JMP version 5.0.1 software (SAS Institute Inc., Cary, NC, USA) and Microsoft Excel 2007. The results were expressed as medians and variations for continuous variables and as numbers and percentages for categorical variables. The differences were considered statistically significant at a significance level of *p* < 0.05.

## 4. Results

### 4.1. Echocardiographic Parameters

**Figure 2:**
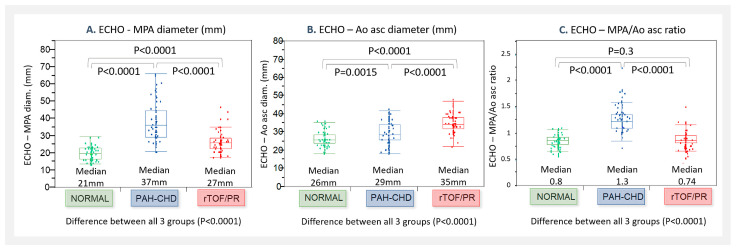
Parameters measured by ECHO, compared in groups (NORMAL, PAH-CHD, and rTOF/PR): (**A**). MPA diameter; (**B**). Ao asc diameter; and (**C**). MPA/Ao asc ratio. NORMAL—control healthy subjects; PAH-CHD—pulmonary artery hypertension associated with congenital heart defects; rTOF/PR—repaired Tetralogy of Fallot with severe pulmonary regurgitation; ECHO—echocardiography; MPA—main pulmonary artery; and Ao asc—ascending aorta.

**Figure 3:**
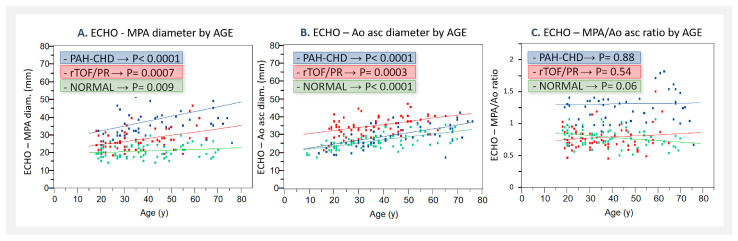
Correlation of parameters by age, measured by ECHO, compared in groups (NORMAL, PAH-CHD, and rTOF/PR): (**A**). MPA diameter; (**B**). Ao asc diameter; and (**C**). MPA/Ao asc ratio. NORMAL—control healthy subjects; PAH-CHD—pulmonary artery hypertension associated with congenital heart defects; rTOF/PR—repaired Tetralogy of Fallot with severe pulmonary regurgitation; ECHO—echocardiography; MPA—main pulmonary artery; and Ao asc—ascending aorta.

ECHO parameters were measured in all three groups as follows:

- **MPA diameter** was significantly greater in PAH-CHD (22–65 mm; median: 37 mm), as well as in rTOF/PR (19–47 mm; median: 27 mm), compared to NORMAL (15–30 mm; median: 21 mm), as shown in Figure 2A. The MPA diameter was increasing with age in all three patients’ groups, as shown in Figure 3A.
- **Ao asc diameter** was significantly greater in PAH-CHD (18–43 mm; median: 29 mm), and even more in rTOF/PR (22–48 mm; median: 35 mm), compared to NORMAL (18–36 mm; median: 26 mm), as shown in Figure 2B. Ao asc diameter was increasing with age in all three patients´ groups, as shown in Figure 3B.
- **The MPA/Ao asc diameter ratio** was significantly greater in PAH-CHD (0.68–2.3, median 1.3), but not in rTOF/PR (0.46–1.52, median 0.74), compared to NORMAL (0.52–1.09, median 0.8)—Figure 2C. MPA/Ao asc ratio did not show statistically significant changes with age in all 3 patients’ groups, as shown in Figure 3C.

### 4.2. CT/MRI Measurements 

All CT/MRI-analyzed parameters also showed significant differences between the PAH-CHD and rTOF/PR groups: MPA diameter was substantially greater in PAH-CHD (33–74 mm; median: 46 mm), compared to rTOF/PR (15–47 mm; median: 30 mm)—*p* < 0.0001; as well as the calculated MPA/Ao asc diameter ratio—in PAH-CHD (1.1–2.42; median: 1.5), compared to rTOF/PR (0.5–1.56; median: 0.94)—*p* < 0.0001. Ao asc diameter was, on the contrary, significantly greater in rTOF/PR (23–46 mm; median: 36 mm), compared to PAH-CHD (22–42 mm; median: 31 mm)—*p* = 0.05.

### 4.3. ECHO and CT/MRI Measurement Comparison

**Figure 4:**
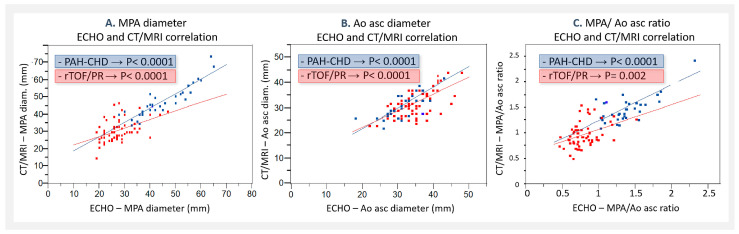
ECHO and CT/MRI correlation: (**A**) in MPA diameter; (**B**) in Ao asc diameter; and (**C**) in MPA/Ao asc ratio. PAH-CHD—pulmonary artery hypertension associated with congenital heart defects; rTOF/PR—repaired Tetralogy of Fallot with severe pulmonary regurgitation; ECHO—echocardiography; CT/MRI—computer tomography/magnetic resonance imaging; MPA—main pulmonary artery; and Ao asc—ascending aorta.

The correlation between ECHO and CT/MRI was found in the overall evaluation (MPA *p* < 0.0001; Ao asc diameter *p* < 0.0001; MPA/Ao asc ratio *p* < 0.0001), as well as in the analysis of MPA, Ao asc, and MPA/Ao asc ratio selectively in both patients’ groups (Figure 4A–C). ECHO, though significantly underestimated in comparison to CT/MRI, was found in all the analyzed parameters (Table 4).

### 4.4. MPA Dilatation—Severity

In PAH-CHD and rTOF/PR, there was a statistically relevant difference in the number of patients with significant MPA dilatation (>40 mm), measured both by ECHO and CT/MRI, as shown in Table 5. The presence of extremely dilated MPA (>50 mm) was only found in the PAH-CHD group—in 16.7% of these patients measured by ECHO and in 31.4% measured by CT/MRI, while no severe MPA dilatation was present in the rTOF/PR group—Table 5.

### 4.5. MPA/Ao Asc Ratio

There was a statistically significant difference in the number of patients with the MPA/Ao asc ratio > 1. In the PAH-CHD group, it was in 88.1% of the patients according to ECHO measurements and in 100% according to CT/MRI measurements, compared to rTOF/PR patients, where it was only in 10.9% according to ECHO and in 34.5% according to CT/MRI measurements, as shown in Table 6.

Severe MPA dilatation (MPA/Ao asc ratio > 1.5) was also present more frequently in PAH-CTD patients (in 25.4% according to ECHO and up to 46.7% according to CT/MRI), compared to a very rare occurrence in rTOF/PR (only in 1.5% of patients according to ECHO and in 1.6% according to CT/MRI)—Table 5.

## 5. Discussion

PA size is not so frequently analyzed in the literature. There is no clearly defined agreement about PA normal size or the optimal strategy for the use of different diagnostic methods for its visualization. Even more, there might still be quite a lot of open questions about the pathophysiological factors that influence PA size and morphology, as well as the clinically relevant severity of PA dilatation, which represents a valid risk for developing life-threatening complications. 

The discussion can be focused on the following major topics:

- **What is a normal-sized MPA, and what is the optimal tool for MPA measurement?**

In our study, the MPA size in the NORMAL healthy population was median 21 mm, with an upper limit of 30 mm. Most studies consider a normal MPA size of <28.9 mm for males and <26.9 mm for females based on the Framingham Heart Study [1] or the upper limit of 29–30 mm without distinguishing between genders [18,19]. On the other hand, there were also several other studies published with significantly lower or higher upper normal limits, varying from 25–26 mm [16,20] up to 32–33 mm [17,18,21,22,23].

The differences in the established normal MPA diameter may also come from different measurement techniques used. Most of the presented studies (including the Framingham study) were based on CT scans, and even among them were many technical differences (native CT scans versus contrast CT angiography; CT performed with or without ECG-gating, etc.). On the other hand, there are really very few studies based on ECHO measurements [14,20,24]. Our study of the NORMAL healthy population was based on ECHO measurements. We consider ECHO, due to its wide availability and, in most patients, at least moderately good MPA visualization, a sufficient diagnostic method for MPA analysis.

Another question is: how significant may the differences between the standard CT, MRI, and ECHO measurement techniques be? 

In our study, we confirmed a very good overall correlation between ECHO and CT/MRI measurements; though, we also confirmed a significant underestimation of ECHO compared to CT/MRI measurements in all analyzed parameters. This fact could possibly also explain the differences presented in various studies, where the smallest presented MPA values (25–26 mm) were in the ECHO studies, while in the CT studies the upper limits for normal MPA diameter were 29–30 mm or more. To our knowledge, though, outside of our study, there has been no direct ECHO versus CT comparison or analysis published so far.

According to the differences in normal values in various studies and the inconsistency in MPA measurement techniques, this topic should perhaps be addressed in larger/multicentric studies and unified in future imaging guidelines.

- **What are the factors influencing the normal MPA size?**

The correlation of the MPA diameter with the patient’s body constitution is a well-known fact in all comparative studies [4,18,21], though a cut-off value indexing the MPA size to height, weight, or the patient’s body surface area still does not exist. 

In our study, we also found a significant correlation between the MPA diameter and age (in all analyzed groups), which in other studies was not always the case. The explanation may be due to the very wide time span of the analyzed subjects in our study, from adolescence and young adulthood to older age (late seventies), compared to other studies, where usually only mid-aged to older population groups were described [21,24]. The age-related progression of the MPA size in our study may be associated not only with the longer period of pathological hemodynamics present in the patients but also with the natural increase in weight in the population throughout the years, as well as some increase in systemic and pulmonary blood pressure with time.

- **MPA dilatation—what are the differences due to pressure overload (PAH-CHD) and volume overload (rTOF/PR)?**

In our study, a significant MPA dilatation was present in PAH-CHD as well as in rTOF/PR patients. Pathological factors that affect the PA size are very complex and, most probably, are still not completely understood. 

The presence of MPA dilatation in pulmonary hypertension (PH) is described almost invariably in all studies and is even considered diagnostic for PH presence [2,25,26,27]. MPA dilatation is usually found regardless of the type of PH [28,29,30,31], though a direct correlation with PH severity often cannot be confirmed [28,29,32]. In our study, we did not analyze the direct correlation of PAH hemodynamics with MPA diameter, as the hemodynamic PAH assessment in many patients was performed in childhood, years before our current study.

**Figure 5:**
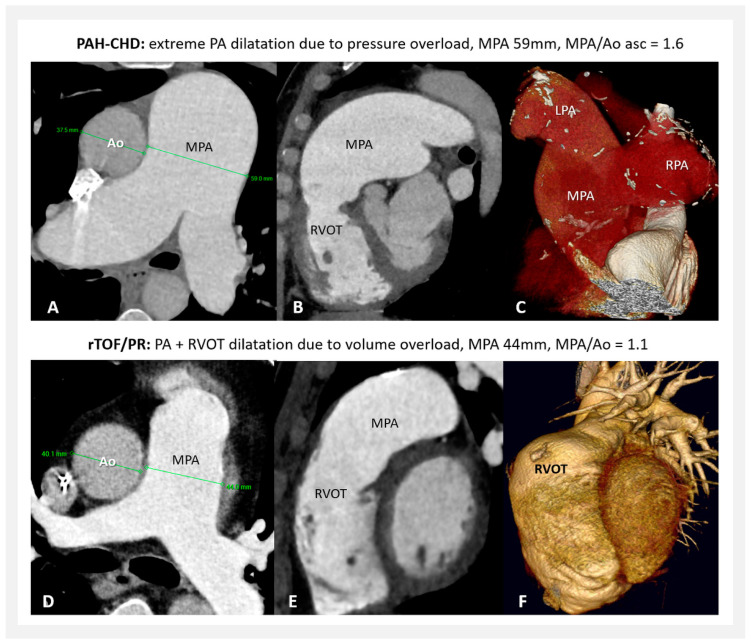
(**A**–**C**) MPA dilatation in PAH-CHD (CT); (**D**–**F**) MPA plus RVOT dilatation in rTOF/PR (CT). PAH-CHD—pulmonary arterial hypertension associated with congenital heart defects; rTOF/PR—repaired Tetralogy of Fallot with isolated severe pulmonary regurgitation; PA—pulmonary artery; MPA—main pulmonary artery; RVOT—right ventricular outflow tract; LPA—left pulmonary artery; RPA—right pulmonary artery; Ao—aorta; and CT—computer tomography.

Analyses of MPA dilatation in accordance with different types of pathological hemodynamic situations are only rarely presented [29,33]. Our study showed a significantly more prominent MPA dilatation in PAH-CHD patients compared to rTOF/PR. According to our data, pressure overload is confirmed to be the most severe underlying pathological process. Above this isolated fact, when the pressure overload is long-term as well as severe, MPA dilatation is continuously progressive with time and can lead to extreme MPA diameters—Figure 5A–C. This was confirmed by our previous study [34].

On the other hand, PH is not the only situation that leads to MPA dilatation. Our study had shown that long-term isolated PA volume overload leads to proximal PA dilatation as well, though not so severe compared to the situation with pressure overload. MPA diameter above the upper normal limit was found in almost half of our patients with rTOF/PR according to ECHO and in more than 2/3 of patients according to CT/MRI measurements. In this patient group, a detailed analysis focused on MPA dilatation was, to our knowledge, not published so far.

The less prominent MPA dilatation due to pulmonary regurgitation may have a more complex explanation. Despite the increased volume load located in the proximal PA, this may increase the vessel wall shear stress less than expected, affecting more the right ventricle (or its outflow tract) instead (Figure 5D,E), but this needs more hemodynamic studies and confirmation. PA turbulence (due to associated stenosis) and/or different antegrade flow patterns may influence the final degree of MPA dilatation as well [33,35,36,37].

- **MPA/Ao asc ratio—how relevant is it and when to use it?**

Not only the MPA diameter itself but also the MPA/Ao asc ratio is considered a very important parameter in defining possible MPA dilatation or, more frequently, in establishing the presence of PH. 

Usually, as pathology is defined the MPA/Ao asc ratio >0.9 [1,2,4,23,38,39], or up to >1.05–1.1 in some studies [3,20]. In severe PH, a high-risk situation for the development of complications is considered, when the MPA/Ao asc ratio exceeds 1.5–2 [8,28,40]. 

In our study, the MPA/Ao asc ratio was significantly higher in the PAH-CHD group but not in rTOF/PR. In PAH-CHD patients, this ratio >1 was found in 88–100% (ECHO—CT/MRI) of our measurements, and even the definition of a high-risk ratio of >1.5 was fulfilled in 25–46% of these patients (ECHO—CT/MRI measurements). We therefore consider the MPA/Ao asc ratio very effective in the analysis of MPA dilatation during the follow-up of PAH-CHD patients.

On the contrary, in our study, the MPA/Ao asc ratio did not show significant relevance in patients with rTOF/PR, as there was no difference found compared to NORMAL. Even in patients with evident MPA dilatation, only in 10–35% (ECHO—CT/MRI) did the ratio reach the level >1. The specific situation here is the underlying pathological development of the heart in the tetralogy of Fallot [41]. Due to the disproportional embryological septation of the conotruncus, there is a significant difference in the size of the aorta and the PA (the PA being smaller and the aorta bigger) not only prenatally/at birth, but this frequently persists throughout life, even despite surgical interventions and different hemodynamic situations years later [42,43,44]. This was also confirmed by our study, where the Ao asc diameter was greater not only compared to NORMAL but also compared to PAH-CHD patients. In this context, we therefore consider the MPA/Ao asc ratio of patients with rTOF/PR absolutely not reliable.

- **What degree of MPA dilatation may be considered high-risk for the development of severe complications?**

The rate of complications related to severe MPA dilatation was not described in this study, but in our previously published data [34]. MPA dissection, thrombosis, or significant coronary artery compression were found in three of our patients in the PAH-CHD group, with MPA diameters of 66 mm, 77 mm, and 65 mm, respectively, at the time of complication occurrence.

There is no clear consensus in the literature regarding the point of clinically relevant degree of MPA dilatation either. Usually, as MPA aneurysm is defined when the MPA diameter exceeds 40 mm, or 43 mm in males [20,40,45]. On the other hand, case reports presenting severe complications related to MPA dilatations (e.g., dissection, coronary artery compression, airway compression, or thrombi) are usually in association with a bigger MPA diameter, above 50–55 mm [8,29,45,46,47]. In some studies, a much more extreme MPA dilatation (>75 mm) combined with a very high mean pulmonary artery pressure (>50 mmHg) is expected to be necessary for the development of life-threatening complications [46].

In our study, severe MPA dilatation (>50 mm) was only present in patients with PAH-CHD—in 16.7% of patients according to ECHO measurements and in 31.4% of patients according to CT/MRI measurements. In our rTOF/PR group (where no patient with Tetralogy of Fallot with absent pulmonary valve syndrome was included), no severe MPA dilatation (>50 mm) was found. The occurrence of MPA diameter > 40 mm in this group was also very rare (in 3.1% of patients according to ECHO and 7.8% according to CT/MRI measurements).

Despite case reports presenting complications usually only in extremely dilatated PA, it is still not clear what MPA diameter represents a real risk for complications and if the risk is only PAH-dependent. We believe that we need to be cautious in all patients reaching the MPA diameter of 50 mm or when fast progression of dilatation (>2 mm/year) during the follow-up is noted [48], even regardless of the presence of PAH. However, it is up to individual consideration when any action due to MPA dilatation is needed. 

## 6. Conclusions

The assessment of the PA diameter is very important and should be included in all standard imaging studies, even repeatedly, during the whole follow-up of the patient. Extreme and progressive PA dilatation, especially when combined with severe pressure overload, is of very high risk for developing life-threatening complications. 

On the other hand, PA dilatation should not be associated only with PH, as other hemodynamic pathologies (i.e., pulmonary regurgitation in patients with Tetralogy of Fallot after correction) contribute to this as well. Therefore, establishing the impact of various pathological situations on PA size, function, and outcome is crucial.

## Figures and Tables

**Table 1:** Patients’ basic characteristics.

Patients’ Characteristics	NORMAL (N = 80)	PAH-CHD (N = 60)	rTOF/PR (N = 64)
Gender: Male/Female	31/50	15/45	27/36
Age (years): median (min.–max.)	34 (18–70)	37.5 (18–76)	32.5 (19–65)
Height (cm): median (min.–max.)	172 (132–193)	158.5 (139–195)	170.5 (150–195)
Weight (kg): median (min.–max.)	68 (32–101)	57 (38–120)	74 (40–108)
BSA (Dubois, m^2^): median (min.–max.)	1.81 (1.1–2.29)	1.57 (1.2–2.4)	1.82 (1.3–2.34)

PAH-CHD—pulmonary arterial hypertension associated with congenital heart defects; rTOF/PR—repaired Tetralogy of Fallot with isolated severe pulmonary regurgitation; NORMAL—without any structural or functional cardiac finding; min.–max.—minimum–maximum.

**Table 2:** PAH-CHD patients´ characteristics.

PAH–CHD: Defect Type	N = 60	%
Unoperated defects/or with significant residual shunts	47	78.3
1. Pre-tricuspid shunt:	13	21.7
- Atrial septal defect, type II;	8	13.3
- Atrial septal defect, type sinus venosus and/or Partial anomalous pulmonary venous repair.	5	8.3
2. Post-tricuspid shunt:	34	56.7
- Ventricular septal defect;	15	25
- Atrio-ventricular septal defect;	9	15
- Persistent arterial duct/or aortopulmonary window;	5	8.3
- Multiple post-tricuspid shunts.	5	8.3
After closure, without/or insignificant residual shunt	10	16.7
Other (congenital portopulmonary hypertension)	3	5
Associated Down’s syndrome	15	25

PAH-CHD—pulmonary arterial hypertension associated with congenital heart defects.

**Table 3:** rTOF/PR patients’ characteristics.

rTOF/PR: Patients’ Characteristics	N = 64
Complete TOF repair—age (years): median (min.–max.)	5 (0.25–21)
- Transannular patch or pulmonary valve tomy: No. of patients (%)	51 (79.7)
- Information about surgery not available: No. of patients (%)	13 (20.3)
Previous shunt palliation: No. of patients (%)	9 (14.1)
- Blalock–Taussig shunt	5 (7.8)
- Modified Blalock-Taussig shunt	1 (1.6)
- Watterston–Cooley shunt	3 (4.7)
Follow-up after complete repair (years): median (min.–max.)	22 (17–53)

rTOF/PR—repaired Tetralogy of Fallot with isolated severe pulmonary regurgitation.

**Table 4:** Comparison of ECHO and CT/MRI—MPA and MPA/Ao asc ratio measurements.

	Overall			PAH-CHD			rTOF/PR		
Analyzed Parameter	ECHO Median	CT/MRI Median	*p*-Value	ECHO Median	CT/MRI Median	*p*-Value	ECHO Median	CT/MRI Median	*p*-Value
MPA (mm)	26	33	<0.0001	37	46	<0.0001	27	32	<0.0001
Ao asc (mm)	30	33	0.01	29	31.5	0.01	32	35	0.002
MPA/Ao asc ratio	0.86	1.12	<0.0001	1.3	1.5	0.002	0.74	0.94	<0.0001

PAH-CHD—pulmonary artery hypertension associated with congenital heart defects; rTOF/PR—repaired Tetralogy of Fallot with severe pulmonary regurgitation; ECHO—echocardiography; CT/MRI—computer tomography/magnetic resonance imaging; MPA—main pulmonary artery; and Ao asc—ascending aorta.

**Table 5:** Classification of the severity of MPA dilatation (% of patients) as measured by ECHO and CT/MRI, comparison of PAH-CHD and rTOF/PR groups.

	ECHO			C T/MRI		
Severity of MPA Dilatation /% of Patients	PAH-CHD	rTOF/PR	*p*-Value	PAH-CHD	rTOF/PR	*p*-Value
- No dilatation (≤30 mm)	13.3%	56.3%	<0.0001	0	31.3%	<0.0001
- Mild dilatation (31–40 mm)	51.7%	40.6%		22.9%	60.9%	
- Significant dilatation (>40 mm)	35%	3.1%		76.9%	7.8%	
- Severe dilatation (>50 mm)	16.7%	0		31.4%	0	

PAH-CHD—pulmonary arterial hypertension associated with congenital heart defects; rTOF/PR—repaired Tetralogy of Fallot with isolated severe pulmonary regurgitation; ECHO—echocardiography; and CT/MRI—computer tomography/magnetic resonance imaging.

**Table 6:** Occurrence of MPA/Ao asc ratio >1 and >1.5, as calculated according to ECHO and CT/MRI measurements, comparison of PAH-CHD and rTOF/PR groups.

MPA Dilatation	MPA/Ao asc > 1			MPA/Ao asc > 1.5		
Examination Technique /% of Patients	PAH-CHD	rTOF/PR	*p*-Value	PAH-CHD	rTOF/PR	*p*-Value
- ECHO	88.1%	10.9%	<0.0001	25.4%	1.5%	<0.0001
- CT/MRI	100%	34.5%		45.7%	1.6%	

PAH-CHD—pulmonary arterial hypertension associated with congenital heart defects; rTOF/PR—repaired Tetralogy of Fallot with isolated severe pulmonary regurgitation; ECHO—echocardiography; and CT/MRI—computer tomography/magnetic resonance imaging.

## Data Availability

The data underlying this article are available in this article.
